# Noninvasive evaluation of renal oxygenation in children with chronic kidney disease using blood-oxygen-level-dependent magnetic resonance imaging

**DOI:** 10.1007/s00247-020-04630-3

**Published:** 2020-02-15

**Authors:** Fenglan Luo, Yi Liao, Kunhua Cui, Yuhong Tao

**Affiliations:** 1 Division of Nephrology, Department of Pediatrics, West China Second University Hospital,Sichuan University, No. 20, Section 3, Renmin Nan Lu, Chengdu, Sichuan 610041 People’s Republic of China; 2 Key Laboratory of Birth Defects and Related Diseases of Women and Children (Sichuan University), Ministry of Education, Chengdu, Sichuan China; 3 Department of Radiology, West China Second University Hospital, Sichuan University, Chengdu, Sichuan China

**Keywords:** Blood-oxygen-level-dependent magnetic resonance imaging, Children, Chronic kidney disease, Kidney, Magnetic resonance imaging, Renal function, Renal oxygenation

## Abstract

**Background:**

Renal hypoxia is considered a final pathway in the progression of chronic kidney disease (CKD). Blood-oxygen-level-dependent magnetic resonance imaging (BOLD-MRI) has shown merit for evaluating renal oxygenation in adults.

**Objective:**

To investigate renal cortical and medullary R2* values by CKD stage and by renal function index in children with chronic kidney disease.

**Materials and methods:**

Twenty-one children with CKD Stage 1–3, 16 children with CKD Stage 4–5, and 6 healthy volunteers underwent a renal MRI using multigradient recalled-echo sequence with 16 echoes. We measured the R2* values of the renal cortex and medulla on BOLD-MRI.

**Results:**

The cortical R2* value was ranked as CKD Stage 4–5 > CKD Stage 1–3 > healthy controls, and the medullary R2* value was ranked as CKD Stage 4–5 > CKD Stage 1–3. There was no significant difference in the medullary R2* value between CKD Stage 1–3 patients and the healthy controls. There was a positive correlation between the R2* values in the renal cortex (*r=*0.73) and medulla (*r*=0.89), and the serum creatinine level (*P<*0.001), and the renal cortical and medullary R2* values were negatively correlated with the estimated glomerular filtration rate (*r*=–0.71 and *r=*–0.89, respectively; *P<*0.001).

**Conclusion:**

BOLD-MRI might contribute to noninvasive assessment of renal oxygenation in children with CKD in vivo but it did not reflect renal function in our sample.

## Introduction

Chronic hypoxia in the kidneys plays an important role in the development of chronic kidney disease (CKD) [1]. Changes in renal hemodynamics, disorders of cellular oxygen metabolism, and changes in microcirculation can lead to chronic hypoxia in the kidneys, which promotes renal fibrosis and a deterioration of renal function [2]. Therefore, early monitoring of renal oxygenation status and early treatment might be valuable. Oxygen-sensitive microelectrodes or laser Doppler flow probes were used previously to study hypoxia and blood flow in the kidneys in animal models, and the role of hypoxia in kidney injury was confirmed [3]. However, these invasive examinations are not suitable for human research and clinical follow-up.

Blood-oxygenation-level-dependent magnetic resonance imaging (BOLD-MRI) is the only noninvasive method for monitoring renal blood oxygen levels [4]. Deoxyhemoglobin was used as an endogenous contrast agent in BOLD-MRI, and the apparent relaxation rate (R2*) was used as a sensitive indicator to assess changes in tissue oxygen partial pressure. An increase in the R2* values suggests an increase in deoxyhemoglobin and a decrease in tissue oxygen partial pressure. In 1996, Prasad et al. [5] first used BOLD-MRI technology on 1.5-tesla (T) MRI to evaluate the relative oxygen levels of human renal cortex and medulla. The BOLD-MRI findings were highly consistent with the results of invasive microelectrodes used in animal experiments to measure renal cortical and medullary oxygen partial pressure [6].

In recent years, BOLD-MRI has been gradually applied to various kidney diseases, such as ischemic kidney injury, chronic kidney disease, kidney transplantation, ureteral obstruction, diabetic nephropathy and kidney tumors [7]. However, BOLD-MRI is rarely used in children with kidney disease. Only Chehade et al. [8] applied BOLD-MRI to evaluate renal function in children with CKD caused by vesicoureteral reflux. This study performed BOLD-MRI examination of kidneys using 1.5-T MRI in children with CKD to investigate the differences in renal cortical and medullary R2* values among different CKD stages, and the relationship between renal cortical and medullary R2* values and renal function indexes in children with CKD and to provide baseline data for the further use of renal BOLD-MRI in children with CKD.

## Materials and methods

### Patients

This study enrolled 37 children with CKD who were admitted to the outpatient or inpatient clinics of the West China Second University Hospital, Sichuan University (Chengdu, China) between January 2015 and June 2018. CKD was defined according to the diagnostic criteria proposed in the guidelines of Kidney Disease Improving Global Outcomes (KDIGO) of 2012 [9]. Six healthy children served as controls. The healthy volunteers had no urinary system diseases, diabetes, hypertension or other cardiovascular diseases, and they had not taken any vasoactive drugs. Their serum creatinine, urinary routine and abdominal MRI results were normal. We used the following exclusion criteria: (1) contraindications for MRI, (2) inability to breath-hold, (3) obstructive nephropathy as suggested by imaging examination and (4) poor image quality and serious artifact interference. Our institutional ethics board approved this study and we obtained written informed consent from all participants.

### Magnetic resonance imaging techniques

All children were asked to fast for 8 h and forego liquid intake for 4 h before MRI examination. Before the examination, all children were trained to practice holding their breath for several seconds according to our instructions. During the examination, all children were asked to breathe freely and to hold their breath alternately. A 1.5-T MRI scanner (Achieva Nova Dual; Philips, Best, the Netherlands) was used to perform a coronal BOLD-MRI scan focused on the renal hilum using a gradient-echo echoplanar imaging sequence. Sixteen echo time chain and fast field echo sequences were used. The following scanning parameters were used: field of view 200×282×70 mm; slice thickness 5 mm; number of slices 12; repetition time 400 ms; voxel size 3×3 mm; and flip angle 45°. Each scan was completed as the subjects held their breath.

### Image analysis and data measurement

We used a Philips Extended MR Workspace 2.6.3.5 workstation (Philips, Best, the Netherlands) for image processing and data measurement. R2* values were obtained via software with a region-of-interest (ROI). The following specific methods were used. First, the R2* maps were generated using MRIcro software [10], so the image clearly showed the change in R2* values. The lowest R2* value appears in red, the highest R2* value in blue. Then, the R2* values were calculated by MATLAB (MathWorks, Natick, MA) software. Measurement method: We outlined the cortical regions-of-interest in one area in the upper, middle and lower parts of the renal cortex, avoiding the edge of the kidney and the boundary between the cortex and the medulla. We also outlined the medullary regions-of-interest in one area in the upper, middle and lower parts of the renal medulla, avoiding renal columns and blood vessels. The T2* values of cortex and medulla in the upper, middle and lower parts of the left and right kidneys were calculated. The R2* values were calculated using the formula R2*=1/T2*, and the average values were obtained.

### Clinical data

We recorded the age, height, clinical diagnosis and serum creatinine for each child. The estimated glomerular filtration rate (eGFR) was calculated according to the Schwartz equation and used for CKD staging [11], such that eGFR (mL/min/1.73 m^2^)=K × (height in cm) / creatinine in μmol/L, where the constant K varies with age and gender (K=32.5 for females 1–18 years old and males 1–13 years old, and K=36.5 for males 13–18 years old).

### Statistical analysis

We performed statistical analyses using IBM SPSS Statistics Version 20 (IBM Corp., Armonk, NY). Quantitative values are expressed as means ± standard deviations. After confirming normal distribution of the data, we used a paired *t-*test to compare the differences in renal cortical and medullary R2* values within each group. We used the single-factor analysis of variance (one-way ANOVA) to compare the R2* differences of cortex and medulla among CKD Stage 1–3, CKD Stage 4–5 and healthy control groups, and, if needed, we performed post hoc multiple comparison of each group using the Student-Newman-Keuls-*q* test (SNK-*q*). Because we did not observe normal distribution for the values of serum creatinine (SCr) and eGFR, we assessed the association between serum creatinine or eGFR, and cortical or medullary R2* values using Spearman rank correlation coefficient analysis. *P*-values less than 0.05 were considered statistically significant.

## Results

### Findings in children with chronic kidney disease

A total of 37 children with CKD and 6 healthy children were included in the study. According to the CKD staging criteria established by KDIGO 2012 [9], children with CKD were divided into the CKD Stage 1–3 (*n*=21) and CKD Stage 4–5 groups (*n*=16). The ages of the 37 children with CKD ranged from 5 years and 8 months to 15 years; the median age was 11 years and 2 months. The ratio of boys to girls was 1.18:1. Neither age nor gender differed significantly among groups (Table 1).

**Table 1:** Clinical characteristics of 43 participants

	Chronic kidney disease Stage 1–3 (*n*=21)	Chronic kidney disease Stage 4–5 (*n*=16)	Healthy control (*n*=6)	*P*-value
Age (years)	10.59±2.63	10.40±2.52	8.74±2.04	0.29
Gender (male/female)	10/11	10/6	3/3	0.70
Serum creatinine (μmol/L)	60.67±29.17	562.09±343.40	33.83±5.88	<0.001
Estimated glomerular filtration rate (mL/min/1.73 m^2^)	132.78±47.81	14.88±6.97	193.71±24.15	<0.001
Underlying disease				
Henoch–Schonlein purpura nephritis	6	3	-	N/A
Lupus nephritis	8	0	-	N/A
Primary nephrotic syndrome	3	2	-	N/A
Chronic glomerulonephritis	0	4	-	N/A
Bilateral hydronephrosis	0	3	-	N/A
IgA nephropathy	2	0	-	N/A
Alport syndrome	0	1	-	N/A
Vesicoureteral reflux	1	0	-	N/A
Unknown	1	3	-	N/A
Cortical R2* value	11.61±0.42	13.00±1.35	10.75±0.18	<0.001
Medullary R2* value	16.00±0.83	21.14±1.90	15.30±0.71	<0.001

Data are expressed as a number (number of cases, gender and underlying disease) or means ±standard deviations

*N/A* not applicable

**Fig. 1:**
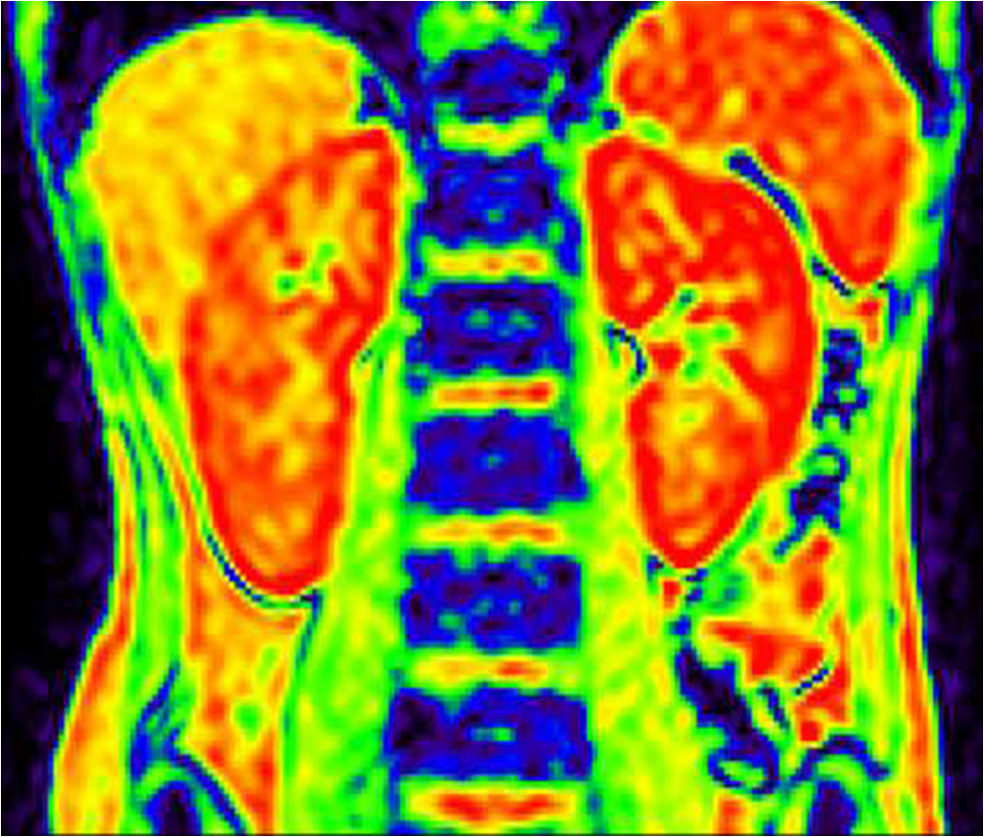
Coronal R2* map of a healthy control (female, 11.2 years old). Blood-oxygen-level-dependent magnetic resonance imaging (BOLD-MRI) map shows increasing R2* values corresponding to gradually decreasing tissue oxygenation levels from red, yellow, green to blue. Here, the normal kidneys appear well oxygenated

**Fig. 2:**
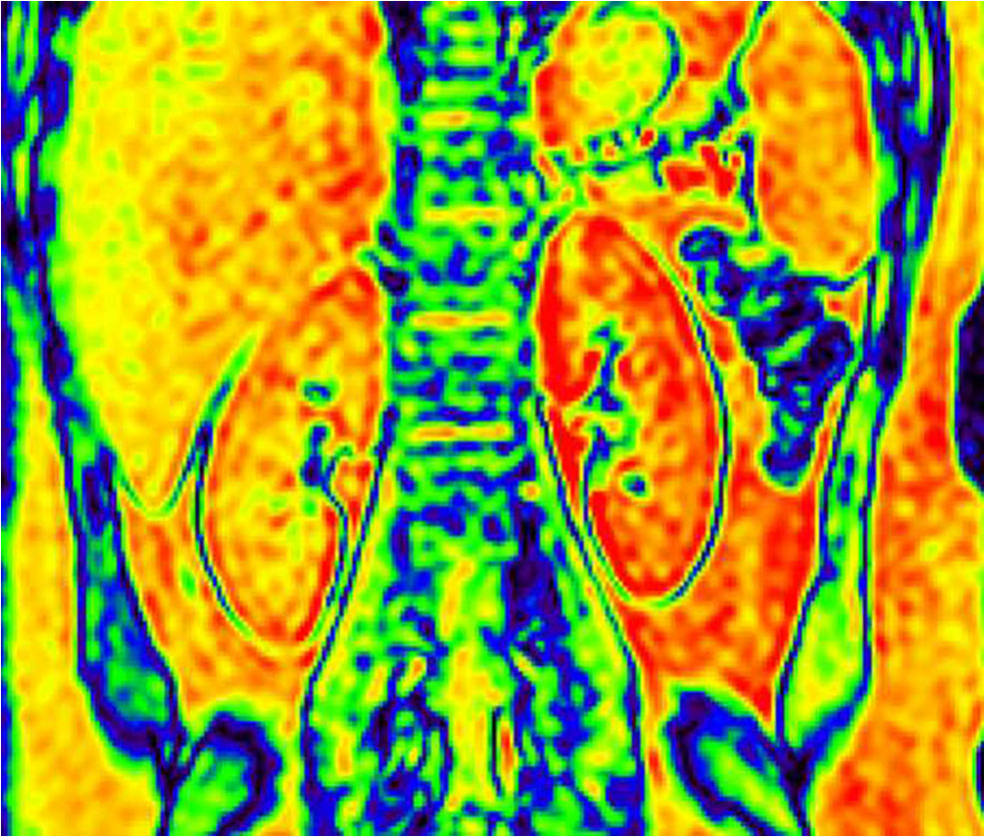
Coronal R2* map in child with moderate chronic kidney disease (female, 12.3 years old). Blood-oxygen-level-dependent magnetic resonance imaging (BOLD-MRI) map shows increasing R2* values corresponding to gradually decreasing tissue oxygenation levels from red, yellow, green to blue. Relatively reduced oxygen level is seen in the right kidney

**Fig. 3:**
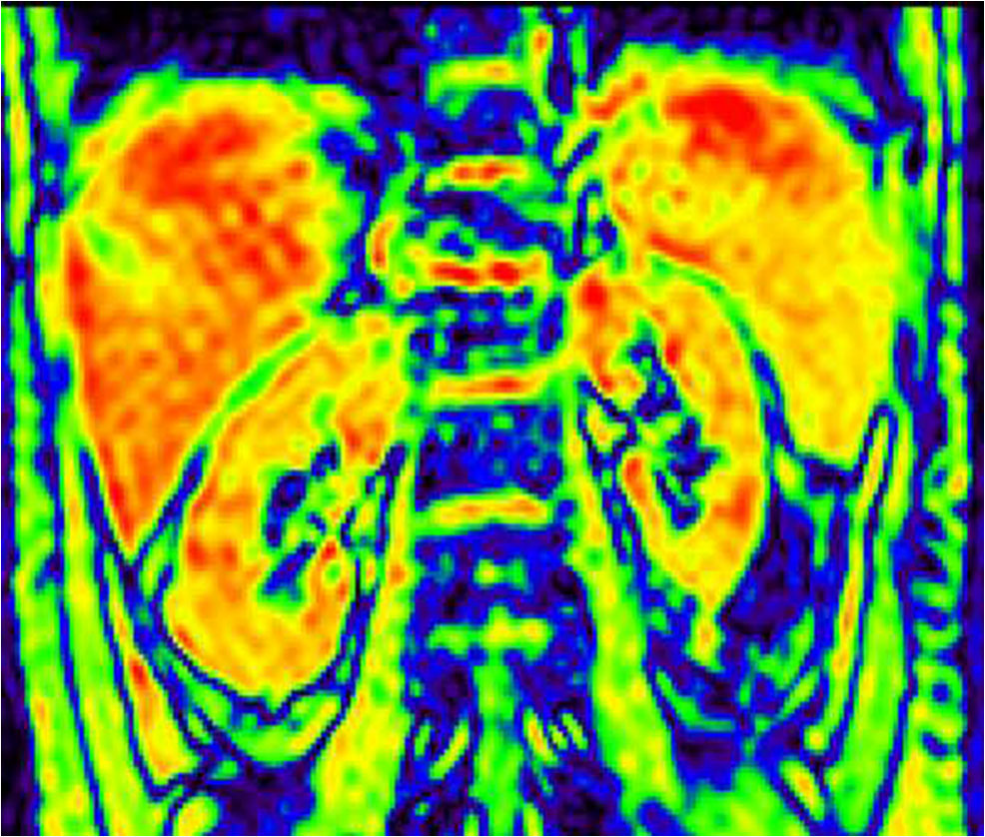
Coronal R2* map in child with severe chronic kidney disease (female, 11.8 years old). Blood-oxygen-level-dependent magnetic resonance imaging (BOLD-MRI) map shows increasing R2* values corresponding to gradually decreasing tissue oxygenation levels from red, yellow, green to blue. Reduced oxygenation is demonstrated in both kidneys

The R2* maps of the renal cortex to medulla of each group showed a color band distribution, which was represented as the cortex in red, and the medulla in yellow, green and blue from the outer to the inner (Figs. 1, 2 and 3). Children with CKD Stage 1–3 and healthy children had a well-defined kidney contour and a clear boundary between the cortex and medulla. Children with CKD Stage 4–5 had reduced kidney volume and unclear boundary between the cortex and medulla.

### Comparison of renal R2* values among groups

**Fig. 4:**
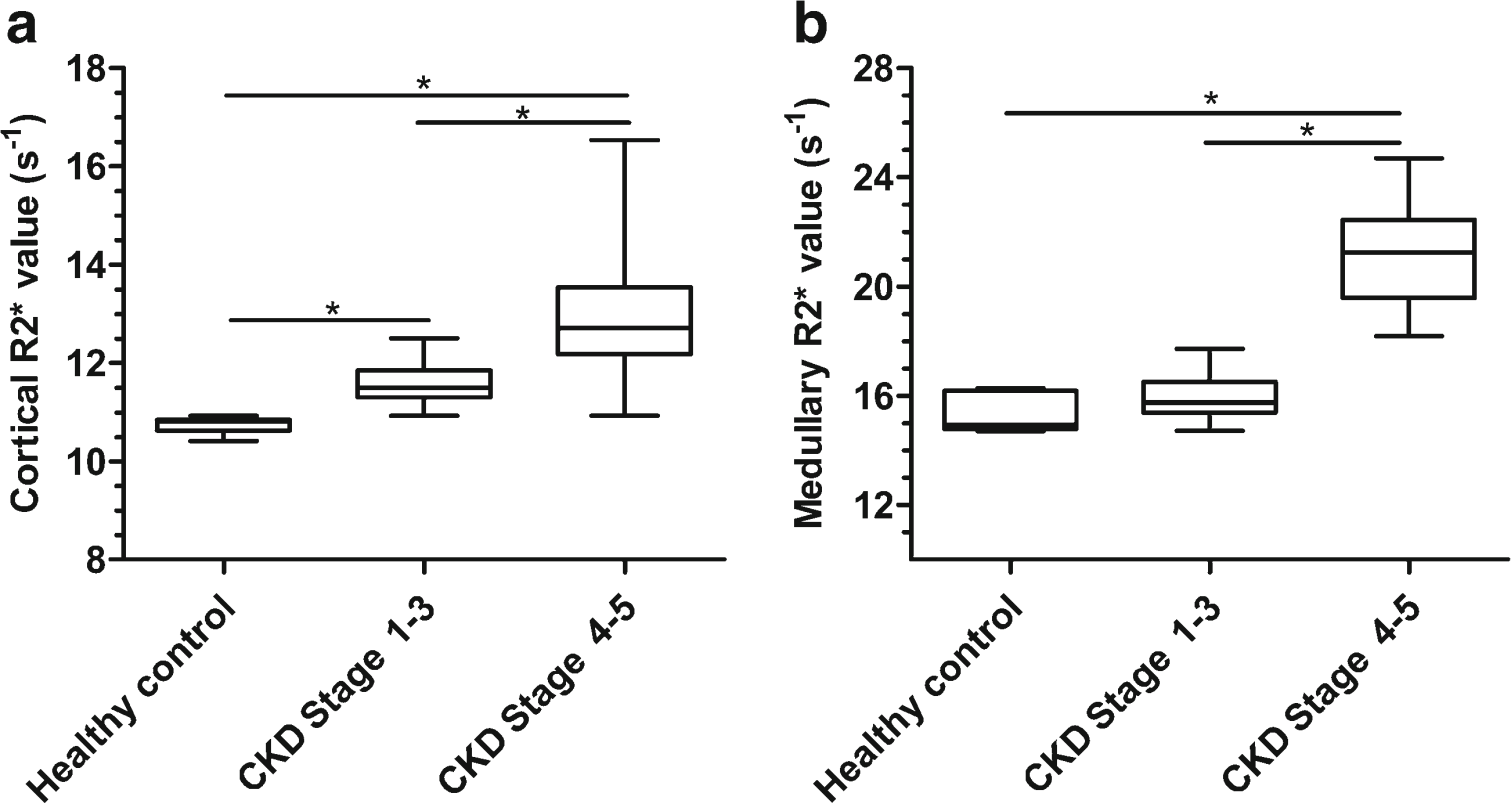
R2* by grade of chronic kidney disease (CKD). **a**, **b** Cortical and medullary R2* values. The cortical R2* values (**a**) are significantly different among CKD Stage 1–3, CKD Stage 4–5 and healthy control groups (*P*<0.01). Medullary R2* values (**b**) of the CKD Stage 4–5 group are significantly different from the other two groups (*P*<0.001)

Because the difference in R2* values between the left and right kidneys was not statistically significant, this study used the mean R2* values of the left and right kidneys to represent the renal cortical and medullary R2* values for each child. The medullary R2* values among the CKD Stage 1–3, CKD Stage 4–5 and healthy control groups were significantly higher than cortical R2* values (*P<*0.05). The cortical R2* values were in the order of CKD Stage 4–5 > CKD Stage 1–3 > healthy controls, and the medullary R2* values were in the order of CKD Stage 4–5 > CKD Stage 1–3. The difference was statistically significant. However, there was no significant difference in the medullary R2* values between the CKD Stage 1–3 and healthy control groups (Fig. 4).

### Correlation between renal function index and renal R2* values

**Fig. 5:**
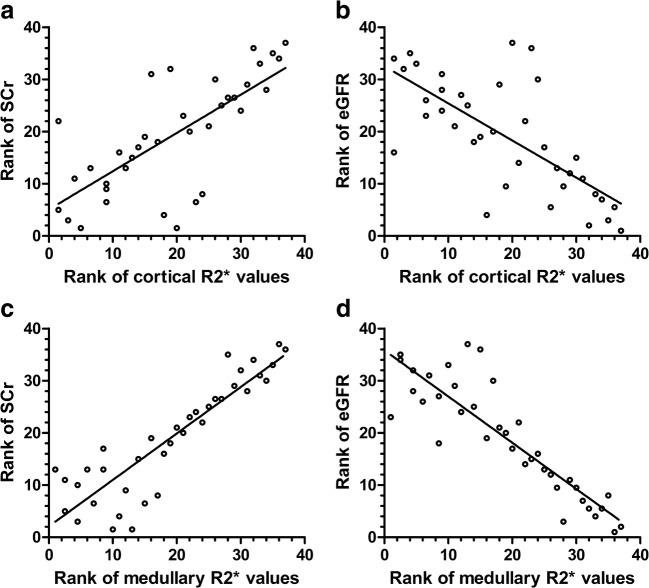
Relation between serum creatinine (SCr) or estimated glomerular filtration rate, (eGFR) and cortical or medullary R2*. **a**, **b** Serum creatinine (**a**) and estimated glomerular filtration rate (**b**) by cortical R2*. **c**, **d** Serum creatinine (**c**) and estimated glomerular filtration rate (**d**) by medullary R2*. Both cortical and medullary R2* values are positively correlated with serum creatinine (*r=*0.73 and *r=*0.89, respectively, *P*<0.001). Both cortical and medullary R2* values are negatively correlated with estimated glomerular filtration rate (*r=*–0.71 and *r=*–0.89, respectively, *P*<0.001)

As shown in Fig. 5, there was a positive correlation between the renal cortical R2* values and serum creatinine in children with CKD (*r=*0.73, *P<*0.001), and the renal cortical R2* values negatively correlated with eGFR (*r=*–0.71, *P<*0.001). There was a positive correlation between the renal medullary R2* values and serum creatinine in children with CKD (*r=*0.89, *P<*0.001), and the renal medullary R2* values negatively correlated with eGFR (*r=*–0.89, *P<*0.001).

## Discussion

We have shown that there was no significant difference in R2* values between bilateral renal cortex and medulla, and that the medullary R2* values of children with CKD and healthy controls were higher than that of the cortex. Our results are consistent with previous studies in adults with CKD and in animals [12–15], which suggests that the renal medulla is in a hypoxic state compared to the cortex, and is also consistent with renal physiology and differences in blood flow distribution between the cortex and medulla. Renal parenchymal perfusion was approximately 25% of cardiac output, and the ratio of cortical and medullary perfusion was approximately 9:1; that means approximately 90% of blood flow was supplied to the cortex, and only 10% was supplied to the medulla. A large amount of oxygen is needed to maintain renal medullary function. The renal cortical and medullary oxygen partial pressures are approximately 50 mmHg and 10–20 mmHg, respectively [16]. However, the R2* values of renal cortex and medulla in the present study were lower than those in adults in the studies of Chehade et al. [8] and Prasad et al. [17]. This difference might be related to the younger age of the participants in our study. Simon-Zoula et al. [18] demonstrated a correlation between the values of the cortex and medulla and age in healthy volunteers, and the values of cortex and medulla gradually increased with age.

Changes in the oxygenation state of the kidney reflect the dynamic regulation between local oxygen supply and oxygen consumption in the kidney. The factors affecting renal oxygen supply include systemic factors, such as renal local blood flow, renal fibrosis, intrarenal microcirculation, and anemia. The factors affecting renal oxygen consumption include glomerular filtration rate, tubular reabsorption function, and Na^+^-K^+^ pump volume. The results presented here showed that the cortical R2* values were in the order of CKD Stage 4–5 group > CKD Stage 1–3 group > healthy control group, and the medullary R2* values were in the order of CKD Stage 4–5 group > CKD Stage 1–3 group. These results are consistent with the findings of Prasad et al. [17], Inoue et al. [19], Manotham et al. [20] and Xin-Long et al. [21], which suggests that children with CKD have relative hypoxia in the kidney, and the degree of hypoxia increases with the severity of kidney damage. The trend is consistent with the hypothesis of hypoxia proposed by Fine and Norman [1].

However, a previous retrospective analysis of adults with CKD using BOLD-MRI showed a large difference between the results of each study [7]. For example, Djamali et al. [22] believed that renal medullary and cortical oxygenation levels were increased in patients with CKD as a result of kidney transplantation, and Siddiqi et al. [23], Khatir et al. [24], Michaely et al. [25] and Pruijm et al. [26] concluded that there was no significant difference in renal cortical or medullary oxygenation levels between CKD patients and healthy controls. The following reasons might explain these differences in BOLD-MRI results among studies: (1) lack of restrictions on drugs or salt intake before MR imaging, (2) lack of water intake standards before MR imaging, (3) different underlying diseases and (4) lack of consensus of BOLD-MR image analysis technology.

Although the medullary R2* values of the CKD Stage 4–5 group were higher than those of the CKD Stage 1–3 group in the present study, there was no significant difference in the medullary R2* values between the CKD Stage 1–3 group and the healthy controls. This finding suggests some overlap of the medullary R2* values between the children with CKD Stage 1–3 and the healthy controls. The reasons might be related to the following factors: (1) in mild renal dysfunction, the kidney has a certain self-regulation mechanism; (2) with renal parenchyma edema, the increase in water content might lead to the reduction of R2* value; or (3) R2* values should be understood not only as changes in oxygen partial pressure but also as affected by MRI magnetic field strength and uniformity, pulse parameters and physiological data (e.g., pH, temperature, hematocrit, hydration effects, spatial vascular conformation, capacity of plasma, and vasoactive substances) [7, 14].

The study also showed that the renal cortical and medullary R2* values positively correlated with SCr and negatively correlated with eGFR. This result suggests that BOLD-MRI is helpful in the evaluation of renal function in children with CKD. However, whether BOLD-MRI has a promising future in children with CKD must be confirmed in large-sample, multicenter randomized controlled trials.

Our study has the following limitations. First, this study was a single-center study with a small sample size, and the majority of the primary diseases were glomerular disease. Further expansion of the sample is needed to investigate differences in the renal cortical R2* values in children with different etiologies.

Second, the image analysis technique used in the present study was an ROI technology, which is the oldest and most common method. When the kidneys function well, the ROI is easy to place. However, for children with advanced CKD, the placement of ROI is difficult because of the lack of visually distinguishable discrimination between the renal cortex and the medulla. Piskunowicz et al. [27] found that ROI technology was prone to observer-dependent differences. They recommended the 12-level concentric object technique because the R2* values are obtained with lower interobserver variability.

Third, MR imaging of the kidney is affected by respiration, intestinal peristalsis and large blood vessel pulsation, with low signal-to-noise ratio, and the magnetic sensitive artifacts caused by gas in the intestinal cavity also interfere with the imaging of the kidney, which limits the use of BOLD-MRI in the kidney. Some younger children cannot hold their breath during MR imaging, which increases the difficulty of MR image acquisition.

Fourth, this study was based on 1.5-T MRI equipment, and the signal change produced by the BOLD effect is proportional to the magnetic field strength. The BOLD effect of large blood vessels is proportional to the intensity of the main magnetic field. The BOLD effect of small blood vessels is proportional to B_0_^2^. High-field-strength MRI further improves image signal-to-noise ratio and spatial resolution. Therefore, high-field-strength MRI might be more conducive to kidney research, and further evaluation of the values of 3.0-T BOLD-MRI for assessing the oxygenation status in children with CKD is needed.

## Conclusion

BOLD-MRI might contribute to noninvasive assessment of parenchymal kidney oxygenation in children with CKD in vivo, but it still doesn’t reflect renal function well. Although considerable research is required to establish BOLD-MRI of kidneys as a routine clinical examination, this technique would be helpful in future clinical trials to further investigate and define the pathophysiological mechanisms of renal disease progression in children with CKD.
